# Ironsand (Titanomagnetite-Titanohematite): Chemistry, Magnetic Properties and Direct Applications for Wireless Power Transfer

**DOI:** 10.3390/ma14185455

**Published:** 2021-09-21

**Authors:** Jérôme Leveneur, William J. Trompetter, Shen V. Chong, Ben Rumsey, Vedran Jovic, Seho Kim, Murray McCurdy, Emma Anquillare, Kevin E. Smith, Nick Long, John Kennedy, Grant Covic, John Boys

**Affiliations:** 1 Earth Resources & Materials, Geological and Nuclear Science, National Isotope Centre, 30 Gracefield Road, Lower Hutt 5040, New Zealand; b.trompetter@gns.cri.nz (W.J.T.); v.jovic@gns.cri.nz (V.J.); m.mccurdy@gns.cri.nz (M.M.); j.kennedy@gns.cri.nz (J.K.); 2 The MacDiarmid Institute for Advanced Materials and Nanotechnology, Victoria University of Wellington, Wellington 6140, New Zealand; shen.chong@vuw.ac.nz; 3 Robinson Research Institute, Victoria University of Wellington, 69 Gracefield Road, Lower Hutt 5010, New Zealand; nick.long@vuw.ac.nz; 4 Verum Group, 68 Gracefield Road, Lower Hutt 5010, New Zealand; b.rumsey@verumgroup.co.nz; 5 Department of Electrical and Computer Engineering, Faculty of Engineering, University of Auckland, Auckland 1142, New Zealand; s.kim@auckland.ac.nz (S.K.); ga.covic@auckland.ac.nz (G.C.); j.boys@auckland.ac.nz (J.B.); 6 Department of Physics, Boston University, Boston, MA 02215, USA; eanquill@bu.edu (E.A.); ksmith@bu.edu (K.E.S.); 7 Advanced Light Source, E. O. Lawrence Berkeley National Laboratory, Berkeley, CA 94720, USA

**Keywords:** magnetic materials, inductive power transfer, ironsand, titanomagnetite, titanohematite, soft magnetic composite, X-ray Absorption Near-Edge Spectroscopy (XANES)

## Abstract

Ironsand is an abundant and inexpensive magnetic mineral resource. However, the magnetic properties of unprocessed ironsand are often inadequate for any practical applications. In this work, the applicability of ironsand for use as a component in a soft magnetic composite for large-scale inductive power transfer applications was investigated. After magnetic separation, the chemical, structural and magnetic properties of ironsand sourced from different locations were compared. Differences observed in the DC magnetic properties were consistent with changes in the chemical compositions obtained from X-ray Absorption Near-Edge Spectroscopy (XANES), which suggests varying the titanohematite to titanomagnetite content. Increased content in titanomagnetite and magnetic permeability correlated well with the total Fe content in the materials. The best-performing ironsand with the highest permeability and lowest core losses was used alongside Mn,Zn-Ferrite particles (ranging from ∼100 μm to 2 mm) to fabricate toroid cores with varying magnetic material loading. It was shown that ironsand can be used to replace up to 15 wt.% of the magnetic materials with minimal impact on the composite magnetic performance, thus reducing the cost. Ironsand was also used as a supporting material in a single-rail wireless power transfer system, effectively increasing the power transfer, demonstrating potential applications to reduce flux leakage.

## 1. Introduction

Ironsand is an important resource for steel and titanium production but has seen limited application beyond that. For example, New Zealand’s ironsand with its iron content in the form of titanomagnetite and titanohematite also makes it a useful supply for titanium. While this ironsand has been exported and used in New Zealand for steel manufacturing for more than 40 years [1,2], there is still ample research to simplify the processing mechanisms and expand the applications of this inexpensive commodity. Recently, for instance, groups have investigated potentially low-cost methods to effectively reduce ironsands, producing microparticles with an insulating TiO_2_ shell and an iron core for simpler processing [3,4]. Applications requiring large quantities of low-magnetic-permeability materials could benefit from such an inexpensive resource. One such application is Inductive Power Transfer (IPT) for vehicle charging.

The driving range of battery electrical vehicles greatly limits their uptake [5,6,7,8,9]. Rather than deploying more stationary recharging stations, a proposition to resolve this issue is to charge the vehicle as it is driving. While the age-old catenaries or rail solutions can be envisaged and will likely have a continuing role in public transport and long-haul land-based heavy transportation [8,10,11], recent efforts have focused on developing a wireless and contact-less power transfer solution [5,12,13]. Among other competing technologies for wireless power transfer [14,15], IPT is arguably the most promising solution [16,17,18]. Even though IPT technology has been conceptualised for over 100 years [19], it has only recently been widely demonstrated [20]. For in-road IPT specifically, a number of medium- and large-scale systems have now been successfully implemented [21,22,23,24,25]. In-road IPT will likely be exposed to extreme conditions ranging from mechanical stress and vibrations from traffic, to possibly difficult environmental conditions—high seasonal temperature variations and varying moisture levels. Hence, such IPT system will have to be designed to endure or be protected from these conditions. In particular, current magnetic core materials found in conventional IPT system are brittle [26] and may not survive these conditions over the decades-long lifetime of the pad [27,28]. In addition, there are limitations in the amount of flux leakage that are acceptable for such applications—to limit public exposure for instance. This makes removing magnetic core materials altogether difficult [29,30,31,32].

Consequently, new cost-effective materials with suitable magnetic performance and mechanical robustness are needed [33]. Soft Magnetic Composite (SMC) [34,35] materials are a prime candidate for such applications as their magnetic and mechanical properties can easily be optimised by controlling the composition and loading of the filler and matrix [36,37]. Recent approaches have investigated the use of various particle sizes of ferrites and other magnetic fillers in various matrices from polymers to cement (including but not limited to [28,38,39,40]). The use of SMCs in an IPT pad has been successfully demonstrated, yet showing higher losses than with the incumbent ferrite [41]. This suggests the need for different pad designs or different magnetic materials. Many alternative soft magnetic composite materials are being produced that could find useful application in such an in-road IPT system. For instance, high-permeability nanocrystalline flakes and powders in a polymer matrix combine the high permeability properties with low conductivity thanks to the insulating matrix [42,43]. Such Polymer-Bonded Magnets (PBMs) have been investigated for use in mobile applications [26,44], and some commercial products are available [45]. Recently, new commercial products have been proposed for use in secondary circuits [46]. To the best of our knowledge, the cost effectiveness and in-road performance of these solutions are yet to be demonstrated.

This investigation looks into the use of natural magnetic materials as an interesting alternative. Demonstrating the usability of such an inexpensive and abundant resource in this IPT application could drastically reduce the cost of materials for large-scale deployment. As titanohematite’s and titanomagnetite’s magnetic properties vary significantly with their stoichiometry [47], ironsand’s magnetic properties vary widely with their provenance [48], which could limit their application.

This article first provides the reader with a basic understanding of the relationship between the chemistry and magnetic properties of ironsand. This should provide useful information to select the right material composition and guide the postprocessing to further improve the magnetic properties (reduction processes for instance). It then goes on to demonstrate the applicability of using such materials in power transfer applications or as a filler in an SMC material. The best-performing ironsands were selected from various geographical locations based on their direct current and alternative current (DC and AC) magnetic properties [48]. Their magnetic properties were analysed to identify the possible optimum cost-effective concentration. By including an explanation of the ironsand chemistry, we show how different ironsand compositions might affect other properties. Toroidal cores were then produced by mixing ironsand within a resin matrix and further characterised for AC magnetic permeability. Different mixes were produced using various concentrations of ironsand and MnZn -Ferrite particles.

## 2. Materials and Methods

### 2.1. Materials

A subset of samples was selected from a series of ironsand samples collected from various New Zealand locations presented previously [48]. This subset represents the samples with the lowest and highest transition metal compositions available. The ironsand samples studied here were the magnetically separated fraction from natural sands with no further processing. The samples’ compositions are provided in Table 1. Prices and costs are indicated in New Zealand dollars. For the chemical characterisation, a magnetite nanopowder was used as a reference (20–30 nm Fe${}_{3}$O${}_{4}$ with >98% purity from SkySpring Nanomaterials, Inc., Houston, TX, USA).

### 2.2. Chemical Characterisation

A previous investigation using ion beam analysis provided the elemental distribution of these samples [48]. The chemical state of the transition metals was investigated for these samples using soft X-ray Absorption Spectroscopy (XAS), and the data were collected at Beamline 8.0.1 of the Advanced Light Source (ALS) synchrotron at the Lawrence Berkeley National Laboratory (LBNL), Berkeley, CA, USA. XAS data were collected in surface-sensitive (∼10 nm depth) total electron yield (TEY) mode by measuring the sample drain current and in the bulk-sensitive (∼100 nm depth) using Total Fluorescence Yield (TFY) mode. The energy scales of the XAS spectra were calibrated with reference to the Ti L-edge and O K-edge spectra of rutile TiO_2_. Spectral resolution was ∼200 meV at Full-Width at Half-Maximum (FWHM). Agreement between TEY and TFY spectra indicated that the samples did not charge during the measurements. The incident X-rays were at 45${}^{\circ }$ relative to the surface normal of the sample. The analysis chamber pressure was below 7×10 ^−9^ mbar during the measurements. The photon energy was calibrated against the values of the O K-edge of anatase TiO${}_{2}$ at 531.3 eV (T${}_{2g}$) and 633.9 eV (e${}_{g}$) [49]. The TEY spectra were normalised against the beam intensity and then normalised again so that the flat pre-edge segment of the spectra was at 0 and the maximum peak intensity was set at 1. A linear background was further subtracted from the Fe L-edge spectra. This signal processing was chosen to easily compare the near-edge features of the Fe L${}_{3}$-edge.

### 2.3. Magnetic Characterisation

The temperature dependence of the magnetisation was also measured between 25 K and 300 K at 1 T using Vibrating Sample Magnetometry (VSM) on a Lake Shore VSM 735 (Lake Shore Cryogenics Inc., Westerville, OH, USA). After a demagnetising sequence, the samples were cooled to the lowest temperature in a 1 T magnetic field, and the magnetisation was measured as the samples were brought back to 300 K. The samples were weighed prior to the measurements to calculate the magnetic moment per mass and the magnetic moment per mass at saturation $M_{s}$. The magnetic moment is reported here in emu·g${}^{-1}$, which is a convenient unit for comparison with the existing literature ($\left[\mathrm{emu}\cdot g^{-1}\right]\left[A\cdot m^{2}\cdot {\mathrm{kg}}^{-1}\right]$, and the Bohr magneton is $\mu_{B}=9.27400994\left(57\right)\times 10^{-21}\;\mathrm{emu}$) [50]. $M_{s}$ was also normalised against the measured concentration of Fe ([] in wt.%) in the samples following:

(1) $$\begin{array}{c} M_{\text{s},\mathrm{norm}.}=\frac{M_{s}}{\left[\mathrm{Fe}\right]} \end{array}$$

### 2.4. Toroid Fabrication

The powder samples were initially packed into 3D-printed moulds. The dimensions of the toroid containers were of height h=9.0 mm, inner radius a=4.5 mm and outer radius b=8.0 mm. The packing density was calculated from the volume of the mould and density of titanomagnetite (5.1 g·cm^−3^) and found at ∼50 vol.%. To investigate the effect of adding magnetic loading on the magnetic properties of the toroid, the EPO-TEK 301 resin (Epoxy Technology, Billerica, MA, USA was used as the binding matrix. EPO-TEK 301 is a two-component (4:1) room-temperature-curing epoxy featuring very low viscosity. Empty toroid moulds were also wound as toroids and used as a reference “air core” toroid.

### 2.5. Complex Permeability Characterisation

The AC magnetic properties were measured by measuring the inductance of an inductor built by winding a 10-turn coil around these toroids. The series inductance values were measured using a handheld LCR measurement unit at 100 kHz. The effective complex permeability was determined using [34]:

(2) $$\begin{array}{c} \mu =\mu^{\prime }-j\mu^{\prime \prime } \end{array}$$

where the real and imaginary parts of the permeability can be calculated from the measured effective complex impedance of the toroid $Z_{\mathrm{meas}}$ and the theoretical or calculated value of the complex impedance of the toroid $Z_{0}$ without a magnetic core (the meas. and 0 subscripts are used hereafter to refer to the values for the measured coil with a core and the “air core”, respectively):

(3) $$\begin{array}{c} \mu =1+[\left(Z_{\mathrm{meas}.}-Z_{0}\right)\frac{2\pi }{\omega \mu_{0}h\times ln\frac{b}{a}}] \end{array}$$

with *a* the outer radius, *b* the inner radius, *h* the height of the toroid and $\mu_{0}$ the vacuum permeability. Consequently, as we can easily measure the series resistance *R* and inductance *L* of the toroid with the magnetic materials and the “air core”:

(4) $$\begin{array}{c} \mu^{\prime }=1+[\left(L_{\mathrm{meas}.}-L_{0}\right)\frac{2\pi }{\omega \mu_{0}h\times ln\frac{b}{a}}] \end{array}$$

(5) $$\begin{array}{c} \mu^{\prime \prime }=[\left(R_{\mathrm{meas}.}-R_{0}\right)\frac{2\pi }{\omega \mu_{0}h\times ln\frac{b}{a}}] \end{array}$$

The loss tangent $\tan\;\delta =\frac{\mu^{\prime \prime }}{\mu^{\prime }}$ was used as a proxy for comparing core losses.

### 2.6. Power Transfer Setup

**Figure 1:**
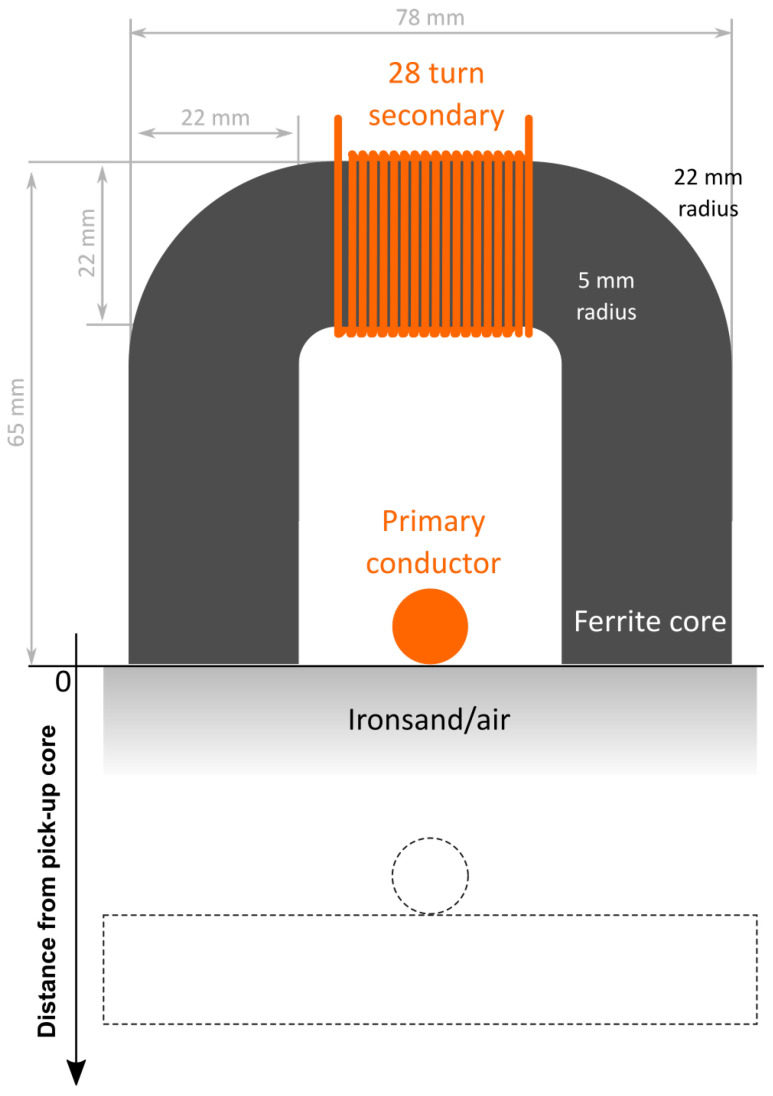
Physical dimensions of the power transfer setup used in this investigation. The dash lines show a different position of the primary circuit with respect to the U-shaped core.

A simple wireless power transfer setup, initially developed to investigate noncontact power transfer for a people mover system [51], monorail systems [52] and Automated Guided Vehicles (AGVs) [20], was used to illustrate the possible usages of ironsand to increase power transfer performance. The primary circuit consisted of a pair of parallel wires, or “tracks”, energised by a low-distortion sinusoidal current. In this investigation, the track current was set at 80 A with a 10 kHz frequency. The primary circuit was maintained constant during all testing. Double-coil designs were investigated for the secondary circuit, called “pick-ups”, making use of a U-shaped soft magnetic ceramic core (Cosmo Ferrite’s CF295) to retrieve power from one or both sides of the primary track. The cores were wound with 28 turns of 1 mm-diameter copper wire around their short side (Figure 1). The open-circuit voltage across the secondary coil and the short-circuit current were measured at successive intervals to determine the untuned transferred volt-amps. This setup allowed varying the distance from the track to the coupled pick-up. Measurements were taken with and without ironsand filling the cores.

A 2D simulation of the system was performed using the Finite Element Modeling Method (FEMM, version 4.2, 2020, MA, USA) software using the physical system’s geometry. The magnetic permeability of the ironsand was approximated as a linear permeability of 1.53 to 4.04 corresponding to the range of ironsand used in the study. A 7-layer Dirichlet boundary condition was applied to the system. The *B(H)* curve of the ferrite from the specifications was used in the model. The magnetic field energy *W* was calculated from the simulated *B* and *H* fields within the block representing the U-shaped core used as the pick-up. This was done using the built-in “Magnetic field energy” block integral function that computes the element-by-element block integral of the product of *B* and *H* over the volume *V* occupied by the core and using the nonlinear *B(H)* curve of the input material, with *dV* a finite volume element [53,54]:

(6) $$\begin{array}{c} W=\int \int_{0}^{B}H\left(B^{\prime }\right)dB^{\prime }dV \end{array}$$

## 3. Results and Discussion

### 3.1. Origin of Magnetic Properties

Table 1 summarises the basic properties of the samples selected for this investigation. As reported previously, the saturation moment of ironsand varies greatly with its provenance and correlates well with the Fe content [48,52,55]. It is also anticorrelated with the content of other elements (denoted as “Crustal” elements in Table 1). Even accounting for the variations in the Fe content, the variations in the magnetic moment remained significant—with a 60% standard deviation of the normalised magnetic moments compared to the initial 48% standard deviation. It is noted that the sample of 4M had a significantly lower magnetic moment per mass of Fe compared to the average of the other samples even though it had a similar Fe content to the most magnetic samples. Samples of 19M and 20M, on the other hand, displayed a magnetic moment per mass of Fe within 10% of the highest values, even though they had almost half as much Fe. Such different magnetic responses suggest differences in the chemical make-up of the samples.

The complex permeability and associated loss coefficient also reflected the changes in magnetic permeability. It was expected that the losses from the wound copper dominate and small variations in winding length and density would affect the measurements more significantly as the magnetic permeability approaches one, also because the measurements were taken relative to the impedance of the “air core” toroid. This explains the nonsensical negative values for the loss tangent and $\mu^{\prime \prime }$. The losses were indeed lower than with the “air core”, but likely not due to the properties of the various ironsand samples.

**Figure 2:**
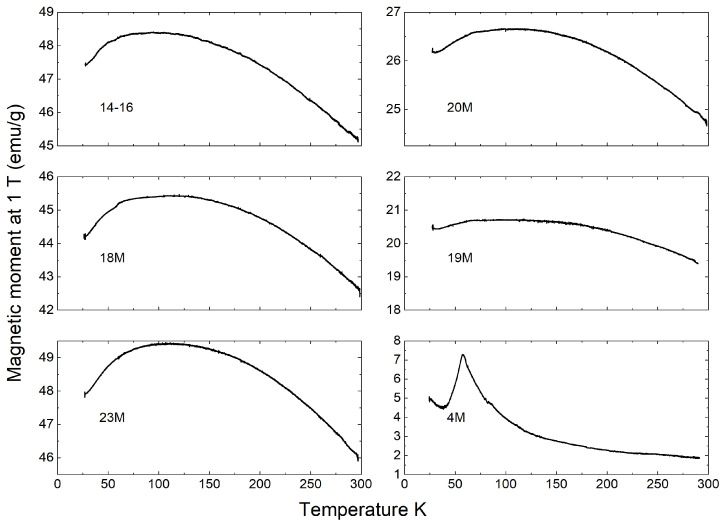
Temperature dependence of the magnetic moment at 1 T obtained with Vibrating Sample Magnetometry (VSM) measurement for all samples as per Table 1. Please note the variation in the scale of the vertical axis.

The temperature dependence of the magnetic moments shed further light on the potential origins for this variation (Figure 2). Indeed, as the magnetic moment became lower, the magnetic state transition became more noticeable around 58 K. This transition was particularly pronounced for the sample of 4M, which had one of the lowest magnetic permeability values. This was consistent with previous observations of magnetic state transitions in the Fe-O-Ti system with high-Ti-content titanohematite or ilmenite (${\mathrm{FeTiO}}_{3}$) [56,57]. Based on other studies of ironsand, it is more likely that this transition originated from titanohematite [3,4].

Understanding the underlying cause for the differences in the magnetic properties presented above can guide the choice of materials and provenance. It can also provide a basis to develop methods to further improve the materials’ performance for a given application. In this context, the chemical structure of each sample can relate directly to its magnetic properties. XAS and X-ray Absorption Near-Edge Spectroscopy (XANES) at the Fe L-edge is a powerful method to differentiate the chemical structure of Fe atoms at the surface of materials. In the Fe-O-Ti system, stoichiometry and ordering significantly affect the energy levels [58]. Indeed, the $L_{3}$ (2p3/2→3d) and $L_{2}$ (2p1/2→3d) transitions are particularly sensitive to the oxidation state and local ordering around the absorbing Fe centre. Taking magnetite Fe_3_O_4_ as a reference, both ${\mathrm{Fe}}^{3+}$ and ${\mathrm{Fe}}^{2+}$ ions are present in a 1:2 ratio, with ${\mathrm{Fe}}^{2+}$ sitting in tetrahedral ($T_{\text{D}}$) sites and ${\mathrm{Fe}}^{3+}$ ions sitting equally in both octahedral ($O_{\text{h}}$) and $T_{\text{D}}$ sites. Variations in the peak intensity corresponding to these transitions inform about the type of oxides present, which can be related directly to the type of magnetic properties that can be expected [58,59,60]. More detailed analysis of the Fe L-edge can be found in the literature [59,61].

**Figure 3:**
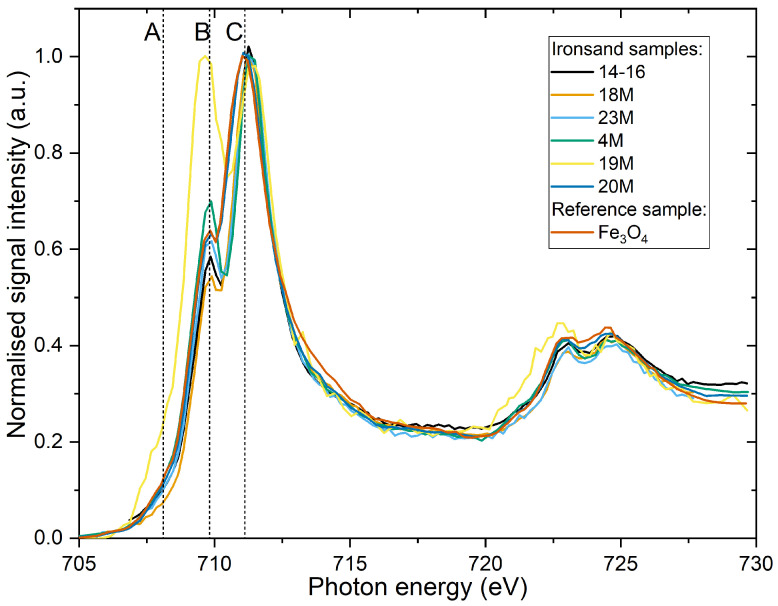
XANES Normalised total electron yield (TEY) Fe L-edge for the different samples and the reference magnetite sample.

As seen in (Figure 3), across the samples, three main features are clearly identifiable at the Fe L_3_-edge around A =708.1eV, B =710.1eV and C =711.1eV. A can be attributed to the presence of Fe in metallic-state ${\mathrm{Fe}}^{0}$ [62], but also can be attributed to multiplets from $T_{D}$-coordinated ${\mathrm{Fe}}^{\mathrm{2+}}$[59,63]. The C and B features are dominated by the response from the ${\mathrm{Fe}}^{\mathrm{2+}}$ ion and ${\mathrm{Fe}}^{\mathrm{3+}}$ ion, respectively. Hence, these absorption edges are typical of mixed iron oxide materials [62].

**Figure 4:**
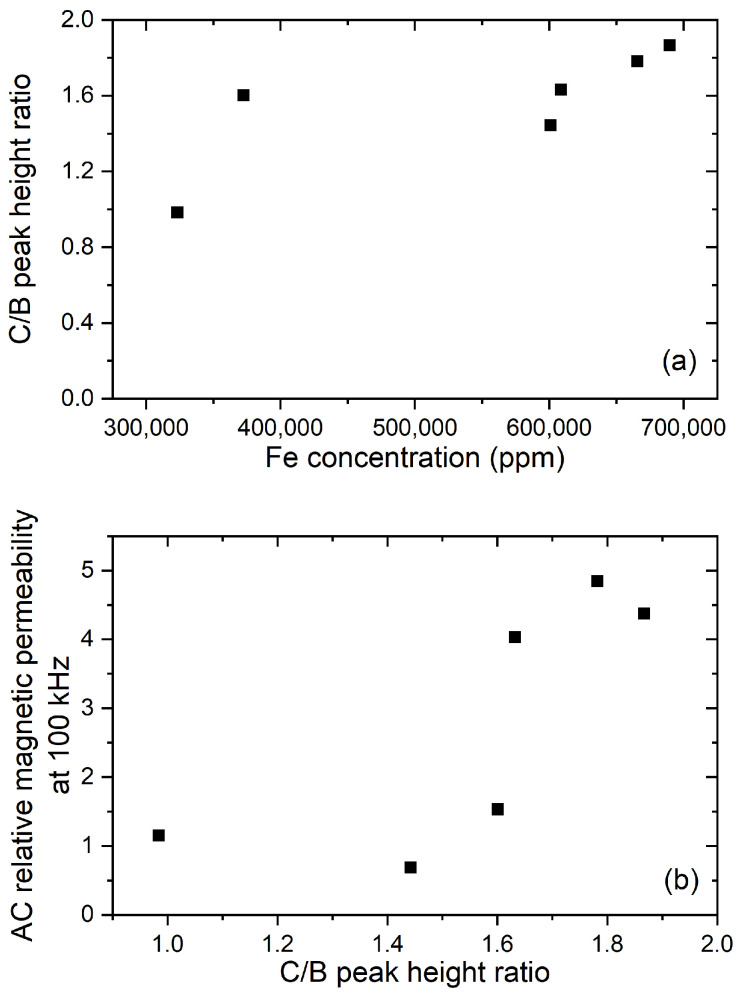
Comparison of the changes of the C/B ratio in the measured Fe L-edges against (**a**) measured Fe concentration and (**b**) magnetic permeability.

Significant differences in the ratio of C to B were observed (Figure 4). Higher C/B ratios were found that were also correlated with a noticeably higher magnetic permeability and magnetic moment at saturation (Figure 4 and Table 1). For comparison, Fe${}_{3}$O${}_{4}$ had a C/B ratio of 1.59. These results were consistent with varying mixtures of titanohematite and titanomagnetite as expected from New Zealand ironsand resources [3,4]. XRD measurements were also consistent with a varying mixture of titanohematite and titanomagnetite (Table S1 and Figures S1–S3 in Supplementary Material). Both higher total Fe content and higher titanomagnetite or metallic Fe concentration are required to have higher magnetic permeability and saturation magnetisation. In addition, for the samples of 23M and 18M, there were two noticeable peaks corresponding to the $L_{2,3}$-edge signal around 778 and 793 eV, but they were not sufficiently resolved to identify the chemistry. These samples were also measured as having some of the highest Co concentrations with XRF and, coincidentally, had some of the highest magnetic permeability. It remains unclear whether the presence of Co traces affects the properties significantly.

### 3.2. Properties of an Ironsand-Based Soft Magnetic Composite

**Figure 5:**
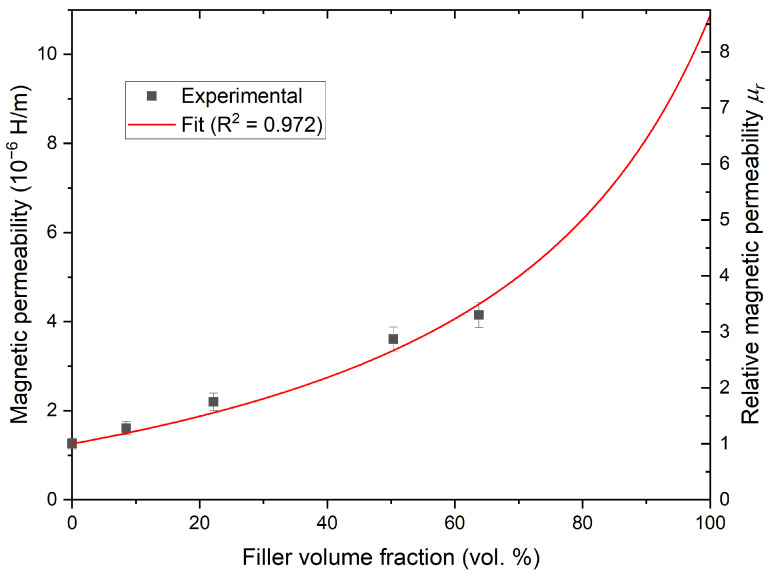
Evolution of the magnetic permeability as a function of the filler volume fraction. The red line is the fit of the experimental data using Equation (7).

Many practical applications may not tolerate the use of loose powders as this might increase variability or be difficult to integrate. It can be more practical to have a bulk solid material. Consequently, the use of ironsand as a base to fabricate a composite material was investigated. The 14–16 material, with the highest magnetic permeability in loose powder, was used in combination with the EPO-TEK-301 resin. The dependence of the magnetic properties of the resulting SMC with various loadings of ironsand were investigated. A higher loading increased the magnetic permeability of the resulting materials (Figure 5). With a loading of 63.8 vol.% (85 wt.%), a relative magnetic permeability of about four was obtained. This value is similar to the value of the ironsand without the matrix, suggesting a similar packing density to the uncompacted powder materials.

The observed trend was consistent with Fricke’s equation to estimate the magnetic permeability μ of a mixture of magnetic grains in a matrix with respective permeability $\mu_{0}$ and $\mu_{1}$ [64,65]:

(7) $$\begin{array}{c} \mu =\mu_{0}[1+\frac{\rho (x+1)}{\left(\mu_{1}+x\mu_{0}\right)/\left(\mu_{1}-\mu_{0}\right)-\rho }] \end{array}$$

where ρ is the volume concentration of the magnetic filler and *x* is an empirical factor. The factor *x* is loosely related to the geometry of the particles [64,65]. This formula successfully fit the experimental values with x=1.9 and $\mu_{r}=8.7$. These values were consistent, within the experimental uncertainty, to the values found in previous investigations of the magnetic properties of ironsands [65].

### 3.3. Combining Ironsand with Higher-Permeability Materials in a Soft Magnetic Composite

**Figure 6:**
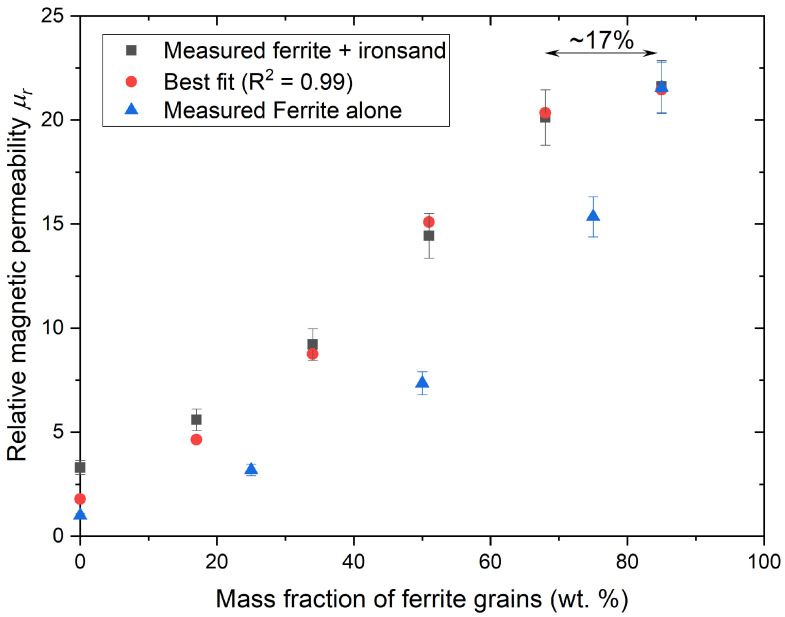
Evolution of the magnetic permeability with different loadings of ferrite and ironsand. The horizontal axis gives the fraction of ferrite grains. The fraction of ironsand is complementary to make a total magnetic filler loading of ∼80 wt.%.

The properties of SMC materials with a combination of ferrite particles and ironsand were measured at different loadings in the same resin. The total mass loading of the magnetic filler was maintained at about 85 wt.% (∼70 vol.%). When comparing the relative permeability of such a composite material, there was no significant difference in the relative permeability at an equal volume fraction of the ferrite particles. However, when comparing the filler loading at an equal mass of ferrite materials, the addition of ironsand to reach an overall magnetic filler fraction of 70 vol.% significantly improved the magnetic permeability of the SMC even though the magnetic permeability of the ironsand + resin composites was significantly lower than the permeability of the ferrite + resin composites (Figure 6). Furthermore, above 40 wt.% magnetic filler, the loss factor of the ferrite + resin and ferrite + ironsand + resin were not significantly different and remained between $7.8\;\mathrm{and}\;8.4\times 10^{-3}$. At a similar loading, the loss factors of ironsand + resin mixes were found around $1.3\pm 0.1\times 10^{-2}$.

These permeability values also fit with Fricke’s equation and assuming the value of the matrix permeability as a resin/ironsand composite. However, the best fit was obtained with different values of *x* and $\mu_{1}$ than what was found previously for composite materials with a single filler (ferrite + resin and ironsand + resin). This suggest that this model and other related models, such as Ollendorff’s [66] or the Clausius–Mossotti formulae [67], are not suitable for the current experimental dataset.

Nevertheless, these results suggest that it is possible to reach the same magnetic permeability with an SMC material where 15 wt.% of ferrite is replaced by ironsand. The main argument in favour of such replacement is the material’s cost. The price of export-grade ironsand is approximately NZD 80 per tonne. Even with initial estimates incorporating the CAPEX and OPEX for milling sand from 400 µm to 100 µm, if needed, this gives a cost per tonne of NZD 88–225 depending on the scale. This is in comparison to the large price tag associated with ferrite particles of NZD 7500 per tonne. However, as the cost of ferrite particles is lowered, so are the cost benefits for using ironsand. Considering an acceptable minimum 10% cost reduction to justify replacing the ferrite by ironsand, the use of ironsand may remain advantageous as long as the cost of ferrite particles remains above NZD 240 per tonne.

### 3.4. Directly Using Ironsand to Increase Power Transfer Efficiency

Placing ironsand to bridge part of an air gap within a magnetic circuit is expected to increase power transfer performance. Indeed, the effective magnetic path can be significantly decreased as the magnetic flux traverses ironsand ($\mu_{r}∼3$) instead of going through air ($\mu_{r}=1$). Hence, such low-permeability SMC can potentially be used to improve the performance of an IPT system by providing an additional layer of control of the flux leakage, particularly where material cost reductions are required. This hypothesis was tested here experimentally by investigating changes in the performance of an IPT setup with and without ironsand.

**Figure 7:**
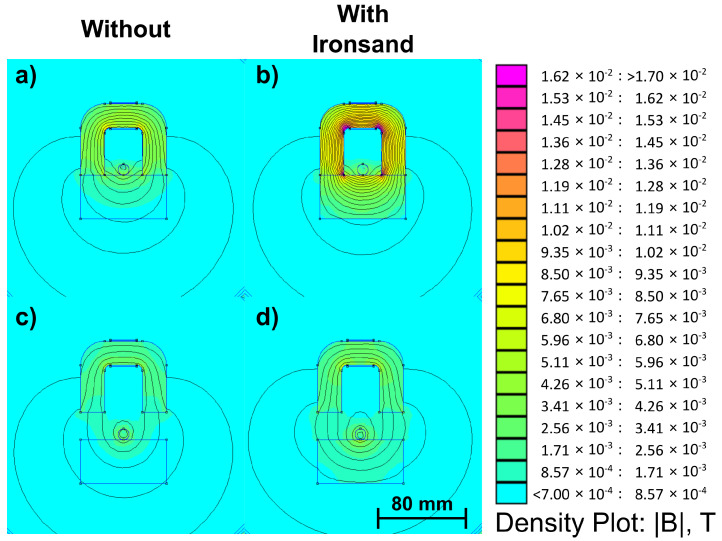
Changes in the magnetic field magnitude with different distances to the pick-up and the presence of ironsand: (**a**) 0 mm, no ironsand; (**b**) 25 mm, no ironsand; (**c**) 0 mm, ironsand; (**d**) 25 mm, ironsand.

A simple IPT configuration was used in this investigation: a primary circuit consisting of a single-rail track was energised with AC current, and a U-shaped core secondary circuit was used as the pick-up. Such systems are typically used to wirelessly power autonomous guided vehicles. Experiments were undertaken to illustrate the benefits of the use of ironsand to reduce the leakage under the track. The rationale was that with a permeability above one, even with values as low as those of ironsand, this would greatly reduce the effective magnetic path length. This can be seen clearly from the simulated field maps (in Figure 7), which showed higher fields in the core when using ironsand. As expected, the benefits of including an ironsand backing quickly dropped as the distance increased.

**Figure 8:**
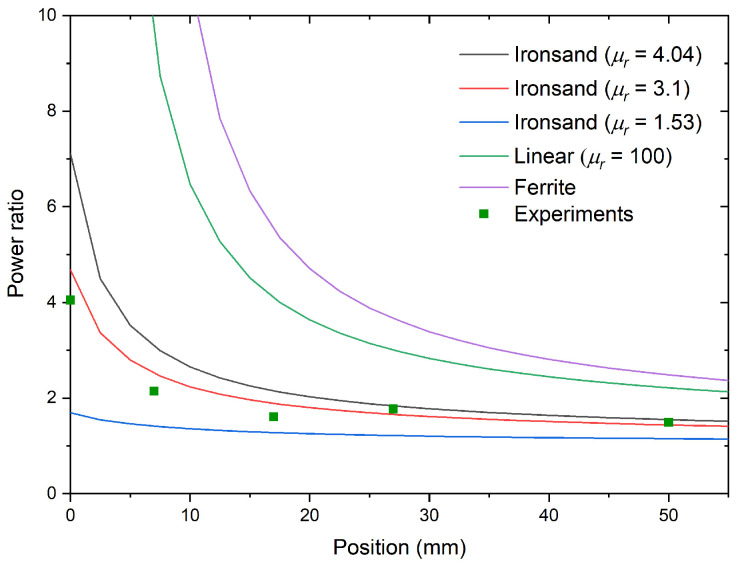
Evolution of the apparent power with/without ironsand at different distances to the pick-up for different relative magnetic permeabilities. The “Linear” dataset corresponds to a hypothetical material that will stay at a constant permeability of 100 over the range of magnetic fields in these conditions. The Ferrite dataset corresponds to the simulation with a slab of the same ferrite and the same thickness as the U-shaped core. The scatter plot represents the experimental values.

Figure 8 gives the ratio of energy transferred to the U-shaped pick-up with ironsand over the energy transferred without ironsand for different distances to the primary circuit. At 50 mm, the transferred power was still about 50% greater than without ironsand. With increasing distance between the rail track and the pick-up core, the benefits of using higher-permeability materials decreased rapidly (Figure 8). At 50 mm, a permeability of 100 increased the power ratio to 52%, whereas using the same ferrite materials ($\mu_{r}∼4000$) only increased this value up to 70%.

While these results did not consider changes in loss by incorporating more magnetic materials in the geometry, they suggest that ironsand and, by extension, other SMC materials could be used practically to further optimise the performance of IPT systems. In particular, despite its low magnetic permeability, ironsand could be used in IPT system designs to further decrease the effective length and to reduce flux leakage. This can be particularly important in large-scale applications where cost is paramount or where stray field exposure needs to be reduced. This includes the in-road IPT systems mentioned in the Introduction.

## 4. Conclusions

It was confirmed that New Zealand ironsand’s magnetic properties were driven primarily by its titanomagnetite and titanohematite content. Increased content in titanomagnetite and magnetic permeability correlated well with the total Fe content in the materials. The presence of traces of Co with higher-permeability ironsand was also confirmed, but its impact on the magnetic properties remains unclear. The magnetic properties of this resource can therefore be selected easily by measuring the total Fe and Co content.

The results obtained with toroids made of an ironsand-based composite allowed the effect of ironsand loading on their inductive properties to be quantified. As expected, a higher loading of ironsand yielded a higher magnetic permeability and a lower loss factor. When combined with larger particles of high-permeability materials, such as a MnZn-Ferrite, it was shown that replacing some of the high-permeability fraction with less expensive ironsand was not detrimental to the magnetic properties. Preliminary cost estimates, based on current commodity prices for ironsand and its basic processing, showed that this could decrease the cost of magnetic materials by 12 to 15%. Further work is also needed to appropriately describe the magnetic properties of such a composite.

Hence, this investigation confirms the potential to use ironsand in an IPT system. This includes using ironsand to replace parts of other higher-permeability-flux-guiding materials without loss of magnetic permeability. Further investigations will focus on the matrix choice and the resulting magnetic, mechanical and thermal properties over the lifetime of an in-road pad. In particular, changes in IPT system designs, including different materials, will have to account precisely for changes in the losses and coupling coefficient.

## Figures and Tables

**Table 1:** Composition and magnetic properties of the ironsand samples used in this study. Sample location can be found in [48].

	Sample	$\mu_{r}$	$\mu^{\prime }$	$\mu^{\prime \prime }$	tanδ	$M_{s}$	*M*_s,norm._	$H_{s}$	Fe	Co	Crustal
					$\left(\mu^{\prime \prime }/\mu^{\prime }\right)\times 10^{-3}$	(emu/g)	(emu/g${}_{\mathrm{Fe}}$)	(Oe)	(ppm)	(ppm)	(ppm)
1	14–16	4.85	4.95	0.03	6.4	50.2	75.4	2564	665,535	2086	89,784
2	18M	4.37	4.46	0.02	5.4	43.2	62.7	2440	689477	3540	136,708
3	23M	4.04	4.12	0.00	1.1	47.2	77.5	2227	608,701	2650	109,485
4	20M	1.53	1.55	−0.02	−1.4	25.4	68.2	2508	372,484	265	452,505
5	19M	1.15	1.15	−0.04	−3.8	19.7	61.0	2591	323,222	942	606,951
6	4M	0.69	0.68	−0.06	−8.3	1.9	3.2	8215	600,871	727	159,545

## Data Availability

Data are available upon reasonable request to the corresponding author.

## Supplementary Materials

The following are available online at https://www.mdpi.com/article/10.3390/ma14185455/s1: Table S1. summary of particle size, saturation magnetisation at 1 T, and XRD identification for different ironsand samples in the study, Figure S1. comparison of the XRD pattern of the 35M samples with the ironsand from previous investigation [4], Figure S2. XRD pattern of the 18M samples, Figure S3. XRD pattern of the 14M samples.

## Abbreviations

The following abbreviations are used in this manuscript:

IPT	Inductive Power Transfer
SMC	Soft Magnetic Composite
XAS	X-ray Absorption Spectroscopy
XANES	X-ray Absorption Near-Edge Spectroscopy
TFY	Total Fluorescence Yield
TEY	Total Electron Yield
FWHM	Full-Width at Half-Maximum
VSM	Vibrating Sample Magnetometry
AGV	Automated Guided Vehicles
FEMM	Finite Element Modelling Method
XRD	X-ray Diffraction
XRF	X-ray Fluorescence
