# Supplementary material for: Ironsand (Titanomagnetite-Titanohematite): Chemistry, Magnetic Properties and Direct Applications for Wireless Power Transfer
Source: Materials (Basel). 2021 Sep 21;14(18):5455. doi: 10.3390/ma14185455 (PMC8466428; doi:10.3390/ma14185455)
Supplement: Supplementary file 1 [file materials-14-05455-s001.zip › materials-1377236-supplementary.pdf]

materials

Supplementary Materials

Ironsand (Titanomagnetite-Titanohematite): Chemistry, Magnetic Properties and Direct Applications for Wireless Power TransferR

Jérôme Leveneur [1,2,*], William J. Trompetter [1], Shen V. Chong [2,3], Ben Rumsey [4], Vedran Jovic [1,2], Seho Kim [5], Murray McCurdy [1], Emma Anquillare [6,7], Kevin E. Smith [6], Nick Long [3], John Kennedy [1,2], Grant Covic [5] and John Boys [5]

[1] Earth Resources & Materials, Geological and Nuclear Science, National Isotope Centre, Gracefield Road, Lower Hutt 5040, New Zealand
[2] The MacDiarmid Institute for Advanced Materials and Nanotechnology, Victoria University of Wellington, Wellington 6140, New Zealand
[3] Robinson Research Institute, Victoria University of Wellington, 69 Gracefield Road, Lower Hutt 5010, New Zealand
[4] Verum Group, 68 Gracefield Road, Lower Hutt 5010, New Zealand
[5] Department of Electrical and Computer Engineering, Faculty of Engineering, University of Auckland, Auckland 1142, New Zealand
[6] Department of Physics, Boston University, Boston, MA 02215, USA
[7] Advanced Light Source, E. O. Lawrence Berkeley National Laboratory, Berkeley, CA 94720, USA
* Correspondence: j.leveneur@gns.cri.nz

This document provides some additional XRD patterns for the various ironsand samples used in this investigations. Powder X-ray diffraction (XRD) analyses were carried out on a Bruker D8 diffractometer using Co Kα radiation (λ = 1.78901 Å) and the operation voltage and current were maintained at 40 kV and 35 mA, respectively. Table S1 summarise the main identified structures while Figures S1 to S3 display the measured patterns.

Table S1. Summary of particle size, saturation magnetisation at 1 T, and XRD identification for different ironsand samples in the study.

Sample Name	Max. Particle Size Distribution Peak (µm)	Saturation Magnetization (emu/g @ 1 T)	XRD Identification
18M	255	43.2	Magnetite, Hematite
11M	153	36.1	Magnetite, Hematite
14M	224	44.0	Magnetite, Hematite
19M	290	19.7	Magnetite, Hematite
20M	329	25.5	Magnetite, Hematite
IPT35M	153	46.4	Magnetite, Hematite

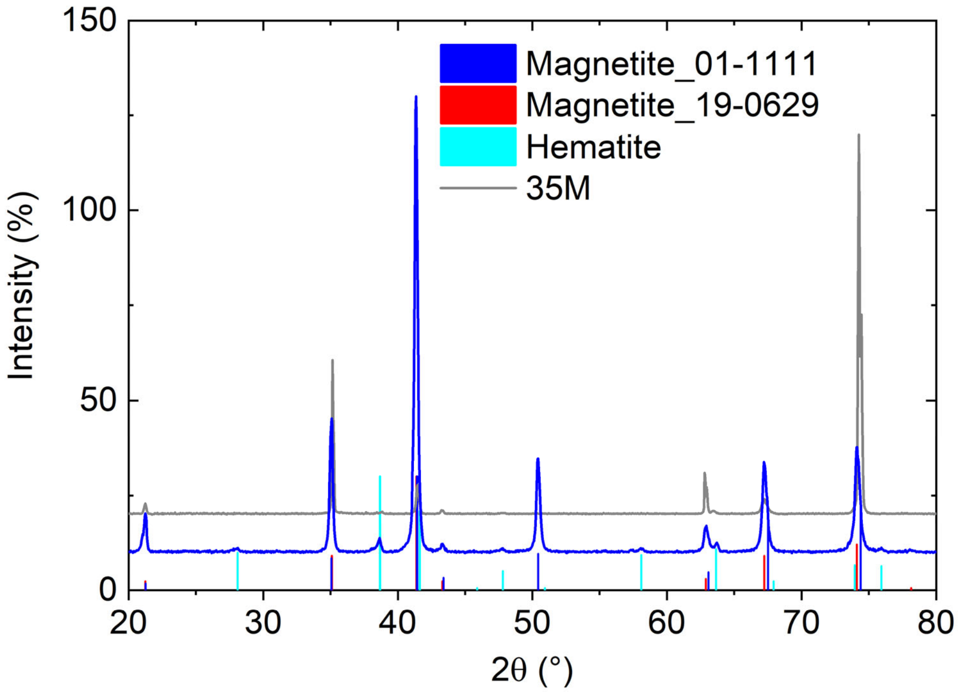

Figure S1. Comparison of the XRD pattern of the 35M sample with the ironsand from previous investigation [1].

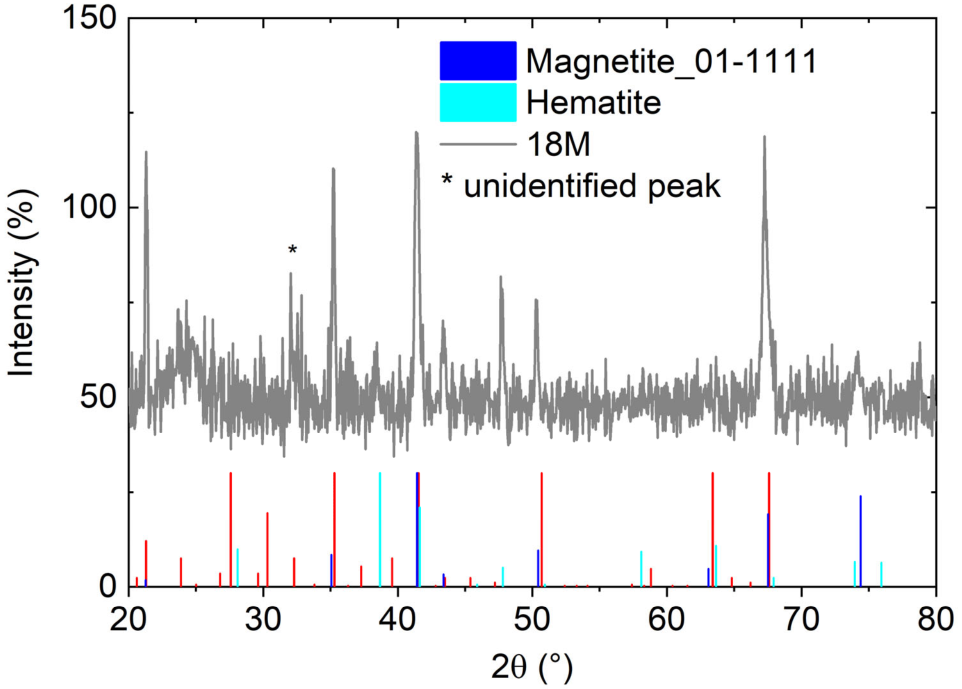

Figure S2. XRD pattern of the 18M samples.

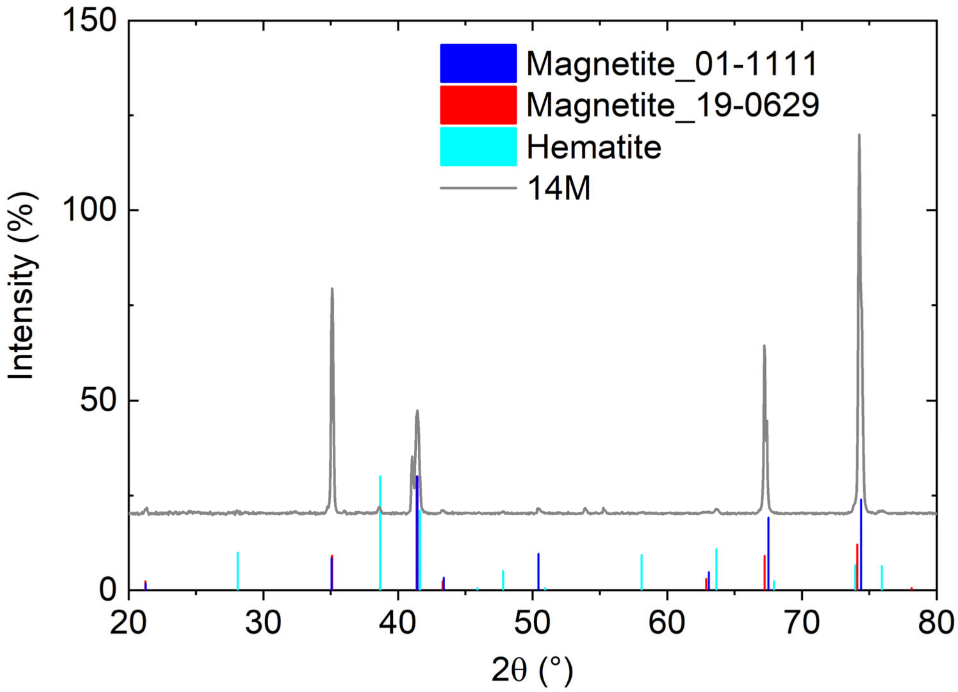

Figure S3. XRD pattern of the 14M samples.

Reference

1. Prabowo, S.W.; Longbottom, R.J.; Monaghan, B.J.; del Puerto, D.; Ryan, M.J.; Bumby, C.W. Sticking-Free Reduction of Titanomagnetite Ironsand in a Fluidized Bed Reactor. *Metall. Mater. Trans. B* **2019**, *50*, 1729–1744.
